# Laser acupuncture-induced analgesic effect and molecular alterations in an incision pain model: a comparison with electroacupuncture-induced effects

**DOI:** 10.1007/s10103-017-2367-7

**Published:** 2017-11-04

**Authors:** Yen-Jing Zeng, Yu-Hsiang Lin, You-Cheng Wang, Ju-Hsin Chang, Jih-Huah Wu, Sheng-Feng Hsu, Shih-Ying Tsai, Ching-Huang Lin, Yeong-Ray Wen

**Affiliations:** 1 Graduate Institute of Clinical Medicine, College of Medicine, China Medical University, Taichung, Taiwan; 2 Microelectronic and Optoelectronic Engineering Department of Electronic Engineering, National Yunlin University of Science and Technology, Yunlin, Taiwan; 3 Department of Electronic Engineering, National Yunlin University of Science and Technology, Yunlin, Taiwan; 4 Department of Anesthesiology, China Medical University Hospital, No. 2, Yuh-Der Rd, North District, 40447 Taichung, Taiwan; 5 Department of Biomedical Engineering, Ming Chuan University, Taipei City, Taiwan; 6 Graduate Institute of Acupuncture Science, College of Chinese Medicine, China Medical University, Taichung, Taiwan; 7 Department of Acupuncture, China Medical University Hospital Taipei Branch, Taipei, Taiwan; 8 Acupuncture Research Center, China Medical University, Taichung, Taiwan; 9 Department of Anesthesiology, School of Medicine, China Medical University, Taichung, Taiwan; 10 Center for Pain Research and Management, Department of Anesthesiology, School of Medicine, China Medical University, Taichung, Taiwan

**Keywords:** Laser acupuncture, Postoperative pain, MAPK, iNOS, TNF

## Abstract

Low-level laser acupuncture (LLLA) produces photobiomodulation through acupuncture point and is an alternative to low-level laser therapy. Although the analgesic effect of LLLA on chronic pain has been proven, its effect on acute postincisional pain has yet to be investigated. A plantar incision (PI) model was used to mimic human postsurgical pain. Male adult rats received GaAlAs laser irradiation at the right ST36 acupoint immediately after operation and on the following 4 days. Three laser treatment groups (two red laser groups with a 30- or 15-min treatment duration and one 30-min near-infrared laser group) were compared with sham LLLA and naive groups and an electroacupuncture (EA) group (separate study). Behavioral withdrawal thresholds of both hind paws were measured before and after incision. Expression of mitogen-activated protein kinases (p-ERK and p-p38), inducible nitric oxide synthase (iNOS), and tumor necrosis factor (TNF) in the spinal cord was analyzed. All three LLLA treatments attenuated post-PI tactile allodynia in the ipsilateral paw, but only the 30-min red laser treatment affected the contralateral paw and had similar efficacy to that of EA. All laser treatments barely reduced heat hyperalgesia in both hind paws. At 3 days after PI, the 30-min red laser group showed reversed increases of PI-induced p-ERK, p-p38, and iNOS but not TNF expression in the spinal cord. Repetitive LLLA treatments ameliorated PI-induced mechanical pain. The inhibition of multiple sensitization signals highlights the unique clinical role of LLLA. Thus, LLLA is an alternative to EA as an adjuvant for postoperative pain control.

**Electronic supplementary material:**

The online version of this article (10.1007/s10103-017-2367-7) contains supplementary material, which is available to authorized users.

## Introductions

Low-level laser therapy (LLLT), recently termed photobiomodulation, involves using a specific range of wavelengths and low power density; it has been widely used for suppressing inflammation, healing wounds, and treating neurological diseases, acute or chronic pain, and degenerative arthritis [1, 2]. Laser beams could also be applied at acupoints as form of acupuncture, termed low-level laser acupuncture (LLLA) [3], with benefits of noninvasive, nonpainful, nonthermal, and noninfectious characteristics [4]. However, no study has assessed the use of LLLA for acute, strong pain.

Surgery is a necessary evil. Poor surgical pain control increases perioperative morbidity and induces chronic postoperative pain [5]. A multimodal analgesic strategy is strongly suggested to reduce opioid-induced side effects [6, 7], and acupuncture or electroacupuncture (EA) has been selected to improve the quality [8]. Some LLLA studies have assessed its application for postoperative pain [3, 9], but these studies were mostly limited to dental, orofacial, and small incision surgeries. Furthermore, whether LLLA exerts equal analgesic effects as LLLT and EA remains unclear.

In this study, we hypothesized that LLLA reduces postsurgical nociception in a rat plantar incision (PI) model [10]. We surveyed incision-induced molecular profiles in the spinal cord to clarify the possible molecular mechanisms to rationalize its clinical use.

## Methods

### Animals

Male Sprague–Dawley rats (230–250 g; BioLASCO, Taipei, Taiwan) were housed in groups of three per cage at a constant 22 ± 2 °C and relative humidity of 40–60% (*v*/*v*); food and water were supplied ad libitum, and a 12-h light/dark cycle was maintained. All experiments were on the basis of the experimental animal “3R principle,” replacement, reduction, and refinement, to minimize number of the animals and performed after approval from the Institutional Animal Care and Utilization Committee, China Medical University, Taichung, Taiwan, in strict accordance with the guidelines for experimental animals [11].

### LLLA and EA

An animal laser stimulation device (Jubilant Sunrise Co., Taiwan), which contains a GaAlAs light-emitting diode laser with four output channels providing two channels of red laser light (wavelength, 650 nm; output density, 1.5 and 3.0 J/cm^2^) and two channels of near-infrared laser light (wavelength, 830 nm; output density, 1.5 J/cm^2^), was used. Laser light was emitted in the pulsed wave mode (15 Hz), with a spot size of 0.03 cm^2^.

**Fig. 1:**
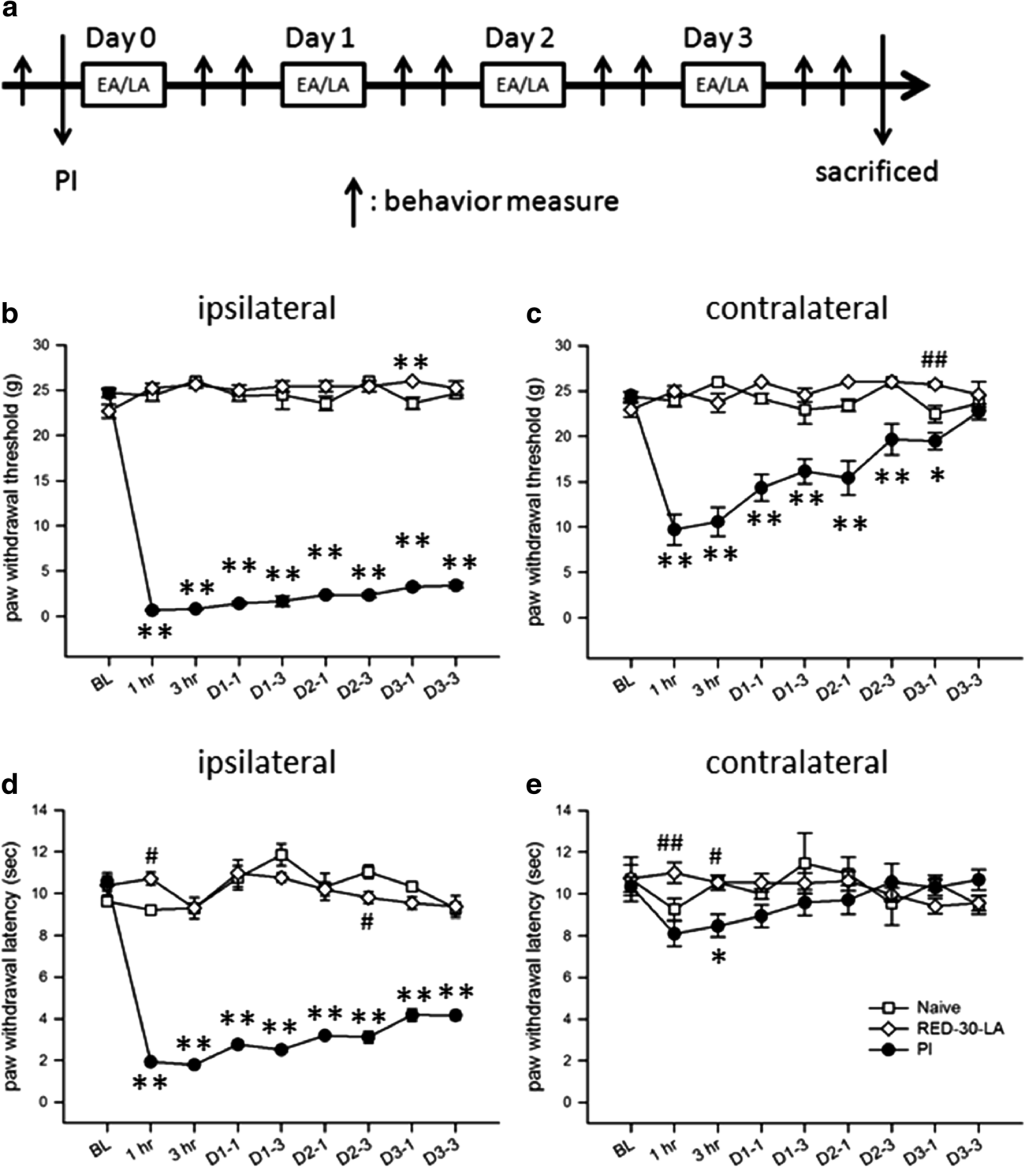
PI-induced bilateral mechanical allodynia and ipsilateral heat hyperalgesia**. a** Diagram of the experiment protocol used in the study. **b**–**e** Nociceptive responses of the naive, PI, and RED-30-LA (i.e., normal rats who received 30-min of RED-LA) groups. **b** and **c** show mechanical withdrawal thresholds in the ipsilateral and contralateral hind paws, respectively, and **d** and **e** illustrate heat withdrawal thresholds in the ipsilateral and contralateral paws, respectively. Abbreviations and symbols: *BL*, baseline data; *D* or *Day*, post-PI day; *D1-1*, post-PI day 1, post-LA 1 h; *EA*, electroacupuncture; *hr*, post-LA hour; *LA*, laser acupuncture; *PI*, plantar incision. Upward arrows mean behavioral measure. *p < 0.05, **p < 0.01 for groups vs. naive group; #*p* < 0.05, ##*p* < 0.01 for groups vs. PI group through one-way ANOVA with Tukey’s post hoc test. No difference was found between the naive and RED-30-LA groups. *N* = 5 (naive), *N* = 6 (PI), and *N* = 5 (RED-30-LA)

The rats were placed in a transparent cylinder holder and were anesthetized with 1% isoflurane gas, as previously described [12]. Both hind limbs were exposed outside the cylinder, and the right hind limb was shaved to expose the skin for irradiation. The laser device was tightly fixed to minimize small movements, and the laser probe was perpendicularly applied at the acupoint (ST36, Zusanli) on the right hind limb. Three types of LLLA beams were applied: red LLLA (RED-LA) for 30 min or 15 min and near-infrared LLLA (NIR-LA) for 15 min. LLLA was conducted immediately after PI (day 0) and repeated for 3 successive days (days 1–3; Fig. 1a).

EA manipulation was conducted using our lab protocol [12]. After stable anesthesia with isoflurane, EA was delivered through a pair of stainless steel needles (36G) inserted at the right ST36. A constant current with square-wave pulses with a 0.5-ms pulse width and 4-Hz frequency was generated by a Grass S88 stimulator and constant current units (Grass, West Warwick, RI, USA). The final stimulation intensity was usually 4–5 mA (about 10 times the muscle twitch intensity) was applied for 30 min.

### PI models

At the right hind paw, a 1-cm longitudinal incision up to the plantaris muscle was made and then sutured. The wound was examined daily, and any sign of wound infection excluded the rat from the study.

To measure the mechanical and thermal thresholds, we performed the up–down method using von Frey fibers (Stoelting, Wood Dale, IL, USA) and the Hargreaves’ test using a glass platform constantly maintained at 30 °C (Plantar Test Apparatus, IITC, CA, USA), as described in our previous study [13]. The experimenter performing the aforementioned two behavioral tests was blinded to the group allocation.

### Western blotting

The rats were euthanized at 3 h and 3 days after PI. The right-dorsal quarter of the L4–L5 spinal cord segment was harvested and frozen in liquid nitrogen. Tissues were homogenized in RIPA buffer (10 μL/mg tissue) containing the appropriate *protease* and phosphatase inhibitors (Sigma-Aldrich, Inc., St. Louis, MO, USA). Equivalent samples (20 μg) were separated on 6–10% SDS–PAGE gel and were electrophoretically transferred to PVDF membranes. After being blocked, the blotting membranes were incubated overnight at 4 °C with polyclonal antibodies against ERK, p-EKR, p38, p-p38 (all 1:1000; Cell Signaling Technology, Danvers, MA, USA), TNF (1:1000; R&D Systems, Inc., MN, USA), iNOS (1:200; Santa Cruz, CA, USA), or GAPDH (1:5000; Novus Biologicals, CO, USA). The blots were then incubated with a HRP-conjugated secondary antibody (Amersham, 1:5000), developed in an enhanced chemiluminescence solution (Millipore, Merck KGaA, Darmstadt, Germany), and exposed onto hyperfilms (Amersham). Specific bands were evaluated with respect to the apparent molecular size and positive control.

### Statistical analysis

All results are expressed as the mean ± standard error of the mean (SEM). Data from behavioral tests were analyzed using two-way analysis of variance (ANOVA). The mean values of western blot analysis were analyzed using one-way ANOVA. Tukey’s post hoc test was employed following ANOVA. Calculations were completed using PASW software for Windows (version 18.0; SPSS Inc., Chicago, IL, USA). *P* values of < 0.05 were considered statistically significant.

## Results

### PI stimulated mechanical hypersensitivity in both hind paws and heat hypersensitivity only in the ipsilateral hind paw

Consistent with our previous studies [14], PI drastically decreased mechanical thresholds from (preoperative) 20–23 g to < 3 g and thermal withdrawal thresholds from 10–12 s to approximately 2 s in the incised hind paw at 1 h (Fig. 1b−e). All rats recovered to a freely moving status within 2 min after the termination of anesthesia, indicating minimized anesthetic influence. Tactile allodynia and heat hyperalgesia in the right hind paw persisted until day 3 after PI. On the contralateral side, tactile thresholds were mildly lowered, indicating mirror-image pain, but heat withdrawal thresholds did not change. Because our data showed that post-PI pain returned to the baseline on day 5 after PI [13], pain behaviors beyond day 3 were not measured.

Notably, the findings of the 30-min RED-LA group did not differ from those of the naive group, indicating that daily irradiation with RED-LA for 30 min did not alter the basal mechanical or heat thresholds.

### Both RED-LA and NIR-LA reduced PI-induced tactile allodynia

**Fig. 2:**
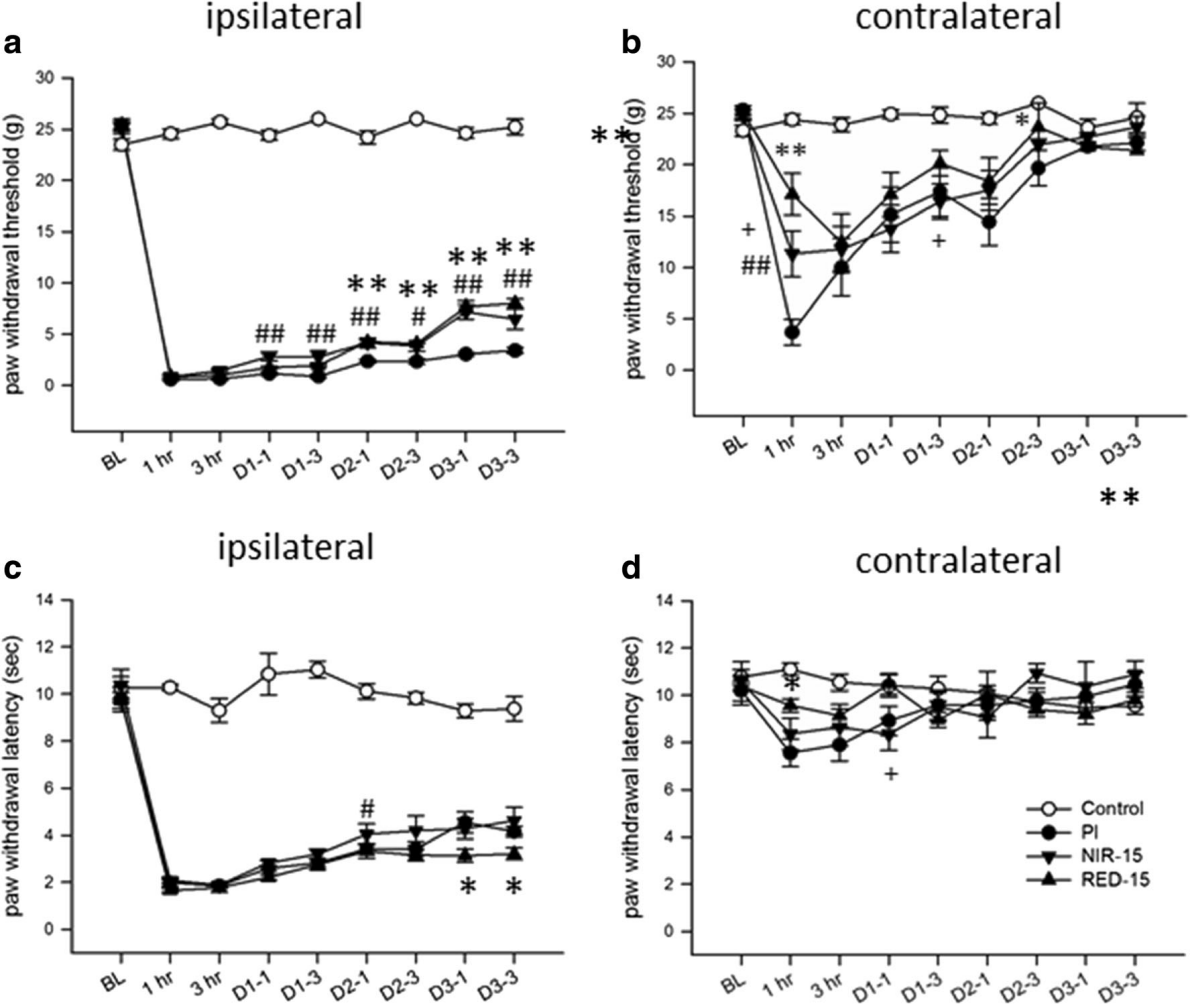
LLLA attenuated PI-induced mechanical allodynia, but not heat hyperalgesia. Comparison of the effects of different wavelengths of LLLA on PI-induced nociceptive hypersensitivity. Red (RED-15) and near-infrared LLLA (NIR-15), which were applied daily for 15 min for 4 days from day 0 after PI, were tested. **a**, **b** Mechanical thresholds. **c**, **d** Heat thresholds. *p < 0.05, **p < 0.01 for RED-15 group vs. PI group, #*p* < 0.05, ##*p* < 0.01 for NIR-15 group vs. RED-15 group, +*p* < 0.05, ++*p* < 0.01 for NIR-15 group vs. RED-15 group through one-way ANOVA with Tukey’s post hoc test. *N* = 5 (control), *N* = 6 (PI), *N* = 7 (NIR-15), and *N* = 7 (RED-15)

RED-LA (650 nm) and NIR-LA (830 nm), LLLA treatments with different wavelengths but the same power density, were applied daily for 15 min to evaluate the impact of wavelength differences. Both treatments attenuated PI-induced mechanical allodynia in the ipsilateral paw from day 1 (i.e., after two cycles of irradiation) but had no effect on heat hyperalgesia (Fig. 2a, c). No differences were observed between the RED-LA and NIR-LA groups; both had analgesia for at least 3 h, but the effectiveness was mild (unpublished lab data). Mirror-image tactile allodynia was reversed by 15-min RED-LA but not by 15-min NIR-LA (Fig. 2b).

### A duration-dependent effect of LLLA on mechanical allodynia

**Fig. 3:**
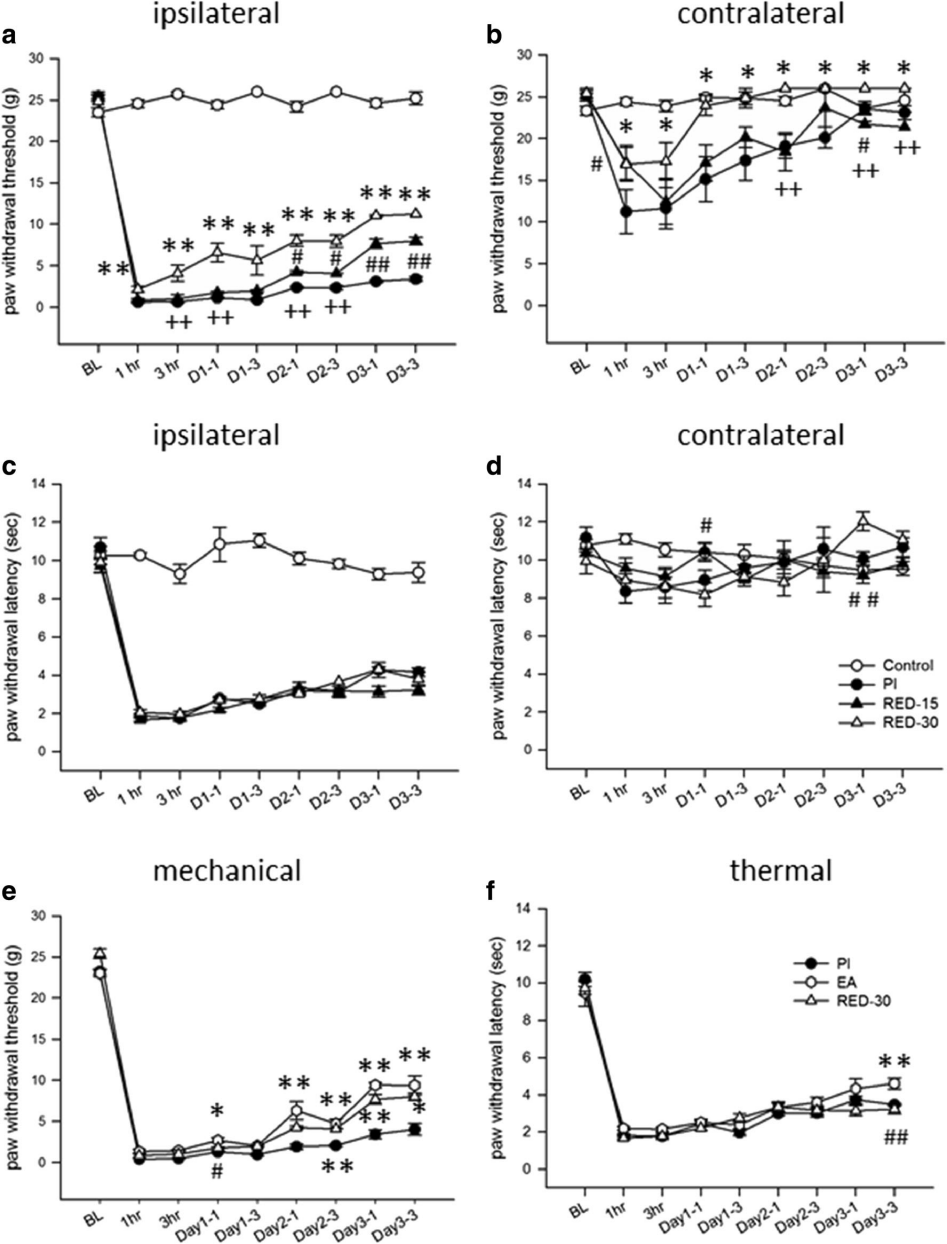
Dose-dependent LLLA analgesic effects and a comparison with EA-induced analgesia. Comparison of the effects on PI-induced nociceptive hypersensitivity between irradiation durations and between LLLA and EA. Two RED-LA durations, 15 min (RED-15) and 30 min (RED-30), were employed. **a**, **b** Mechanical allodynia. **c**, **d** Heat hyperalgesia. **e**, **f** Mechanical and heat hypersensitivity in the ipsilateral hind paw. *p < 0.05, **p < 0.01 for groups vs. PI group, #*p* < 0.05, ##*p* < 0.01 for groups vs. RED-30 group through one-way ANOVA with Tukey’s post hoc test. *N* = 5 (control), *N* = 6 (PI), *N* = 7 (RED-15), *N* = 6 (RED-30), and *N* = 6 (EA)

The irradiation duration influenced the analgesic effect. For both hind paws, the 30-min RED-LA group showed significantly stronger reversal effects on mechanical hypersensitivity than did the 15-min RED-LA group (Fig. 3a, b), indicating that prolonging irradiation from 15 to 30 min enhanced analgesia, with an earlier occurrence of analgesia and a greater accumulating effect. However, no effect was observed on heat hypersensitivity in either paw (Fig. 3c, d), suggesting that LLLA may affect only the mechanical nociceptive pathway.

### RED-LA produced comparable suppression to that produced by EA on PI-induced mechanical allodynia

EA stimulation followed the same time-course protocol as that used for LLLA irradiation (Fig. 3e, f). We noted that 2-Hz EA at an intensity of 4–5 mA for 30 min significantly attenuated PI-induced mechanical allodynia on the PI side (Fig. 3e). The gradually increasing analgesia is very similar to that observed with 30-min RED-LA treatments, both in analgesic efficacy and analgesic duration. In addition, both LLLA and EA had no effect on heat hyperalgesia (Fig. 3c, d, f). Because we aimed to compare the differences between LLLA and EA, there is no control group in Fig. 3e, f. However, we had controlled EA studies in our previous publications [13, 15].

### LLLA significantly inhibited p-ERK, p-p38, and iNOS but did not affect TNF

**Fig. 4:**
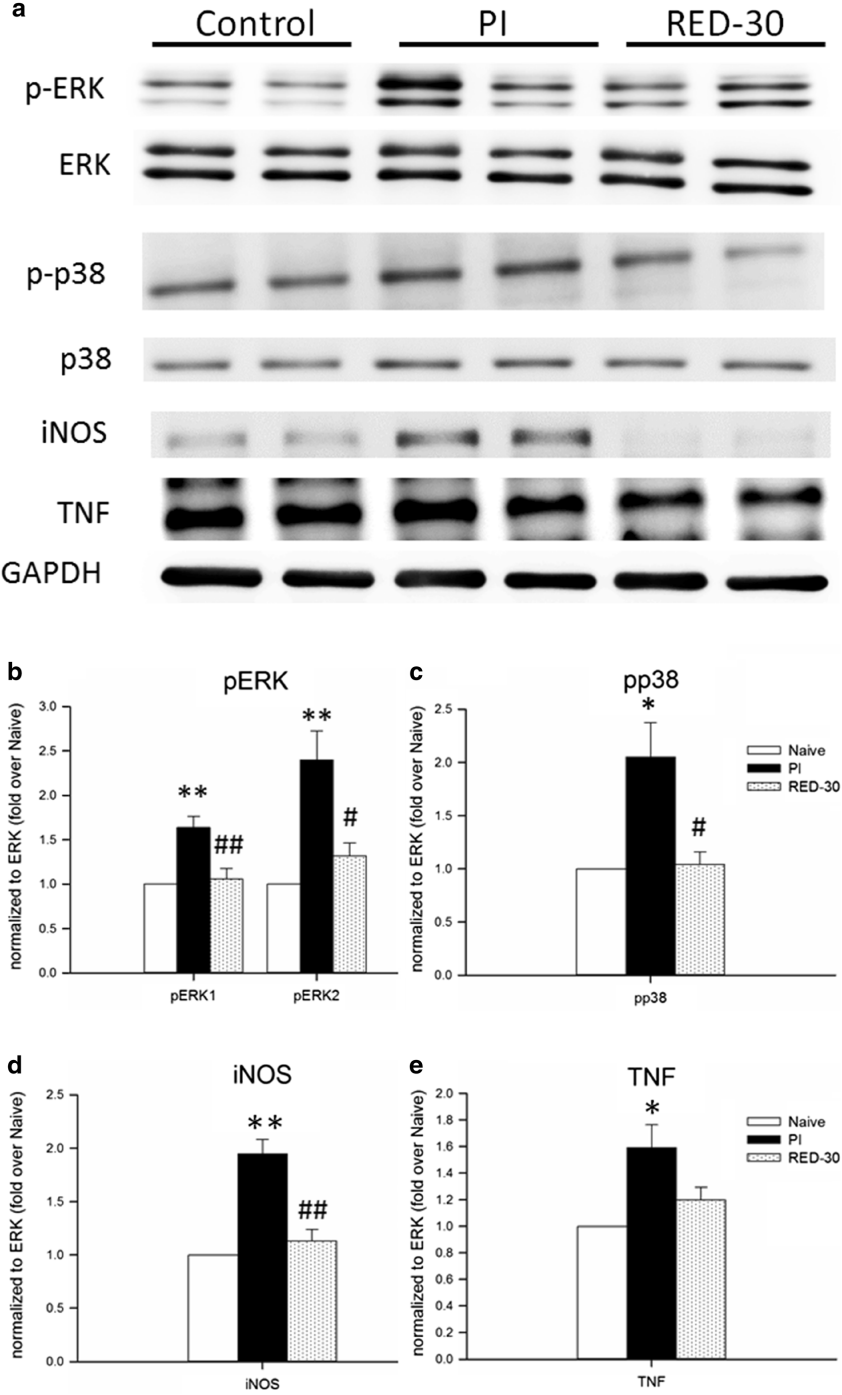
LLLA suppressed PI-induced molecular activation. **a** Representative western blot diagram of protein expression among the groups on post-PI day 3. **b** Relative p-ERK1 and p-ERK2 levels. **c**, **d**, **e** Relative p-p38, iNOS, and TNF levels among the groups, respectively. *p < 0.05, **p < 0.01 for RED-30 group vs. PI group; #*p* < 0.05, ##*p* < 0.01 for RED-15 group vs. PI group, +*p* < 0.05, ++*p* < 0.01 for RED-15 group vs. RED-30 group through one-way ANOVA with Tukey’s post hoc test. *N* = 4–5 for each group

We examined alterations of the spinal dorsal MAPK, TNF, and iNOS expression at 3 h and 3 days post-PI. Western blotting showed that p-ERK, p-p38, iNOS, and TNF levels were significantly increased in the PI group compared with the naive group at 3 days after PI (Fig. 4). In the 30-min RED-LA group, daily RED-LA treatments significantly reduced p-ERK, p-p38, and iNOS expression (Fig. 4b–d). However, LLLA had no effect on TNF expression at 3 days after PI (Fig. 4e). In comparison, expressions of MAPK, TNF, and iNOS in the spinal dorsal horns were not affected by LLLA at 3 h after PI (Supplement Fig. 1).

## Discussion

In this study, LLLA ameliorated incision-induced mechanical hypersensitivity with a comparable effect to that of low-frequency high-intensity EA stimulation. The analgesic effects depended on the duration of irradiation, and the effects accumulated through repetitive application. LLLA reduced MAPK activation and iNOS expression in the spinal dorsal horn, implying postsynaptic analgesic actions of LLLA.

The irradiation dose has been a crucial determinant of LLLA effectiveness in this study and in other studies [3, 16]. We determined that a higher irradiation density, a longer duration, and repeated application improved analgesic effects; however, we do not intend to develop any standard protocol here because additional factors regarding lasers, such as wavelength, power density, pulse mode, and acupoints (single or multiple, unilateral or bilateral, and point combination), should be considered [3].

Both 15-min RED-LA and NIR-LA produced mild suppression of PI-induced mechanical allodynia, but these groups showed no significant difference. The wavelength of a low-power laser falls into an ‘optical window’ between red and near-infrared (600–1070 nm) for maximal penetration. The irradiation depth of this window for the shaved ‘ex vivo’ skin of mice was within 2.5–3.5 mm [17]; therefore, whether LLLA could penetrate to the depth of ST36 in humans is unknown. However, the depth of energy transmission is not only dependent on laser beam profiles but also governed by skin properties, including thickness, age, sex, and inflammation, all could affect outcome.

Increasing irradiation density by prolonging the laser time from 15 to 30 min significantly enhanced analgesia. RED-LA for 15 min provided a radiant exposure of 1.8 J/cm^2^. According to the Arndt–Schultz law [18], biostimulation occurs at doses between 0.05 and 10 J/cm [19], and an optimal value between 0.5 and 4 J/cm^2^ could reduce pain and inflammation. In 2010, the World Association for Laser Therapy recommended a radiant power output of 5–500 mW as a clinically appropriate window for LLLT by using 780–860-nm GaAlAs lasers and suggested a dosage of 4 J per point for plantar fasciitis [20]. Regarding acupoint employment, a study of myofascial pain using 830-nm GaAlAs LA showed positive effects with an at least 10-mW power and 0.5 J/point, whereas those with 0.1–0.2 J/point had negative effects [21]. Because most knowledge regarding the therapeutic window is derived from LLLT application on injured tissues rather than on acupoints, effective doses of LLLT and LLLA for reducing inflammation, nociception, and oxidative stress or for inducing vasodilatation and cellular proliferation [22–24] should to be verified before further study [25].

LLLA application for 4 successive days in normal rats did not cause any alterations in mechanical or heat withdrawal thresholds. This result demonstrates the safety of using such low-energy irradiation on skin, contrary to the risks of EA-related infection or inflammation.

This study also provides a head-to-head comparison between RED-LA and EA. For the first time, we identified that a low power but sufficient dose of laser stimulation at an acupoint could produce an equivalent effect to that of EA on acute postsurgical pain. The two study protocols were very similar, involving the same acupoint, anesthetic procedures, animal manipulation, times and durations of repetitive interventions, and experimenter. Higher LLLA irradiation doses exert a stronger effect, similar to the intensity-dependent EA effect observed by using low-frequency, high-intensity EA (4 Hz, 10 mA) in our previous study [13, 15]. However, both LLLA and EA have low effectiveness. According to our study [13], the EA effect was equipotent to an intraperitoneal injection of morphine at a dose of 1 mg/kg. Altogether, LLLA is not only comparable to EA in reducing postoperative pain but may also be a superior choice for patients with a needle phobia or bleeding diathesis [3].

Furthermore, similarities in analgesic patterns between LLLA and EA imply that they may have analogous mechanisms, despite having distinct physical properties (i.e., laser light and heat vs. electricity and needling pain). LLLA could activate endogenous opioidergic and serotonergic (5-HT1 and 5-HT2A receptors) systems in acetic acid- and formalin-induced nociception (indicating visceral and inflammatory pain, respectively), and the analgesic effects were reversed by naloxone, pindolol, and ketanserin but not by ondansetran [26]; all of these pathways have also been observed in EA analgesia [27, 28]. In addition, recent studies have determined that LLLA activates brain networks to produce a central modulation effect, which is not the same as effects after EA [28, 29].

All three members of the MAPK family (ERK, p38, and JNK) have been hallmarks of nociceptive sensitization in different pain models and play a critical role in nociceptive development and maintenance through distinct pathways within spinal neurons and glia [30, 31]. In a PI model, studies show that the inhibition of spinal p-ERK and p-p38 evidently attenuates PI-induced pain behaviors [14, 31, 32], and activated ERK contributes to the initiation of hypersensitivity immediately after incision [32]. EA pretreatment attenuated PI-induced mechanical hypersensitivity and decreased the number of spinal p-ERK-ir cells distributed in the superficial laminae as early as 30 min after treatment [13]. In this study, daily 30-min RED-LA did not affect early p-ERK levels at 3 h after PI (Supplementary Fig. 1A) but reduced p-ERK expression at 3 days after PI. This result suggests that the LLLA effect is weak and slow, and repetitive laser irradiation may be essential for accumulating analgesia.

A strong EA stimulation (2 Hz, 10 mA, 4 days) activated stronger p-p38 expression than sham EA [15], whereas we observed LLLA significantly decreased p-p38. This implies that LLLA may be less irritating in p38 activation than EA stimulation, while maintains acupoint-mediated antinociceptive action. However, such comparisons may not be accurate because of differences in quantification methods, i.e., immunofluorescence vs. western blot.

NO, after synthesis by NOS, serves as an essential early warning signal and contributes to nociceptive maintenance [33]. In rats, the major source of NO in the spinal dorsal horn is interneurons located in laminae II and III [34]. Different from the definite role of neuronal NOS in sensitizing spinal circuit, the role of iNOS in central transmission remains unclear [35]. Studies have proved that iNOS is required for inflammatory pain [36, 37], and highly selective iNOS inhibitors, 1400W [38] and GW274150 [39], reduced thermal hyperalgesia. Importantly, we firstly report LLLA significantly suppressed iNOS in the spinal cord.

TNF, a proinflammatory cytokine, was expressed in microglia, astrocytes, and primary sensory dorsal root ganglion neurons [40] but rarely in spinal cord neurons [41]. It also plays an essential role in neuropathic pain induced by nerve injury or inflammation [42]. Accumulating evidence suggests that TNF enhances the central mechanisms of neuropathic pain, including c-fiber-evoked long-term potentiation and microglial p-p38-mediated synaptic plasticity [43]. LLLT irradiation at the injured wound could exhibit TNF-related anti-inflammatory properties [44]. The present study demonstrated that although PI significantly increased TNF expression in the ipsilateral dorsal horn, repetitive RED-LA could not reverse increased TNF expression. Nevertheless, the inhibition of spinal microglial p-p38, but not TNF, by LLLA suggests an alternative pathway of microglia-mediated proinflammatory cytokines.

Repetitive LLLA treatments ameliorate incision-induced mechanical pain, whereas the analgesic efficacy is slow and low. In addition, analgesic efficacy of LLLA is analog to that of high-intensity EA, and the inhibition of several sensitizing signals suggests a unique role of LLLA from EA in postsurgical spinal modulation. In conclusion, this preclinical study provides a theoretical basis for the clinical use of LLLA in postoperative pain patients and gives LLLA a novel impetus to become a valuable alternative to EA.

## Electronic supplementary material

Supplement Fig. 1 LLLA did not alter spinal expressions on 3 h post-PI. A, B, C, D: Relative p-ERK1, p-ERK2, p-p38, iNOS, and TNF levels among the groups, respectively. One-way ANOVA with Tukey’s post hoc test and no significant difference among groups. *N* = 4–5 for each group. (GIF 215 kb)

High resolution image (TIFF 124 kb)
