# Supplementary figures and images for: Laser acupuncture-induced analgesic effect and molecular alterations in an incision pain model: a comparison with electroacupuncture-induced effects
Source: Lasers Med Sci. 2017 Nov 4;33(2):295–304. doi: 10.1007/s10103-017-2367-7 (PMC5803293; doi:10.1007/s10103-017-2367-7)

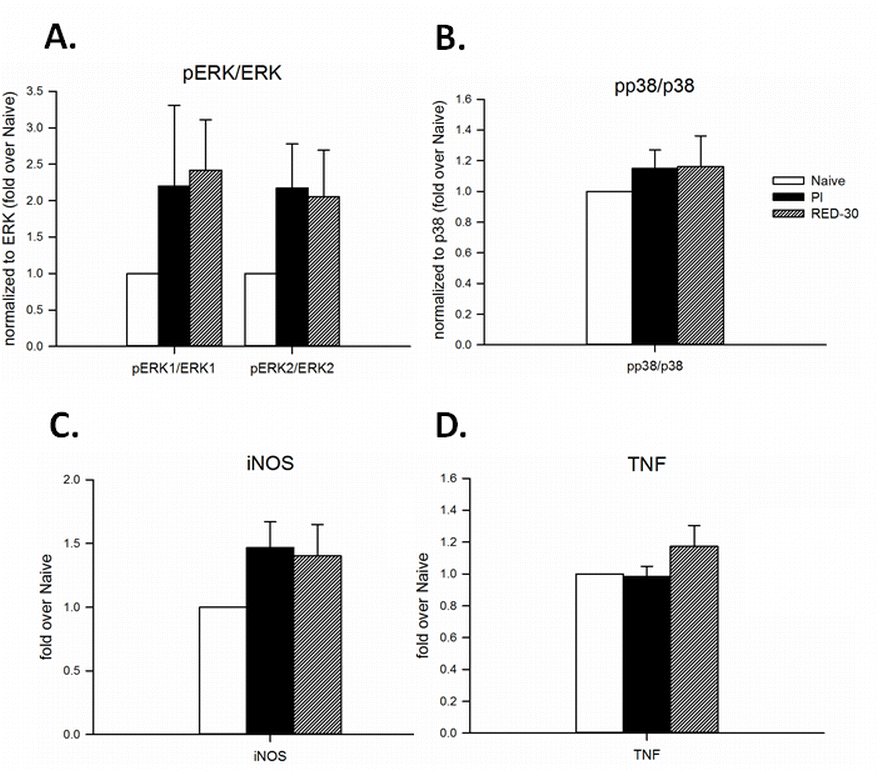

Supplement: Supplementary file 1 — LLLA did not alter spinal expressions on 3 h post-PI. A, B, C, D: Relative p-ERK1, p-ERK2, p-p38, iNOS, and TNF levels among the groups, respectively. One-way ANOVA with Tukey’s post hoc test and no significant difference among groups. N = 4–5 for each group. (GIF 215 kb) [file 10103_2017_2367_Fig5_ESM.gif]

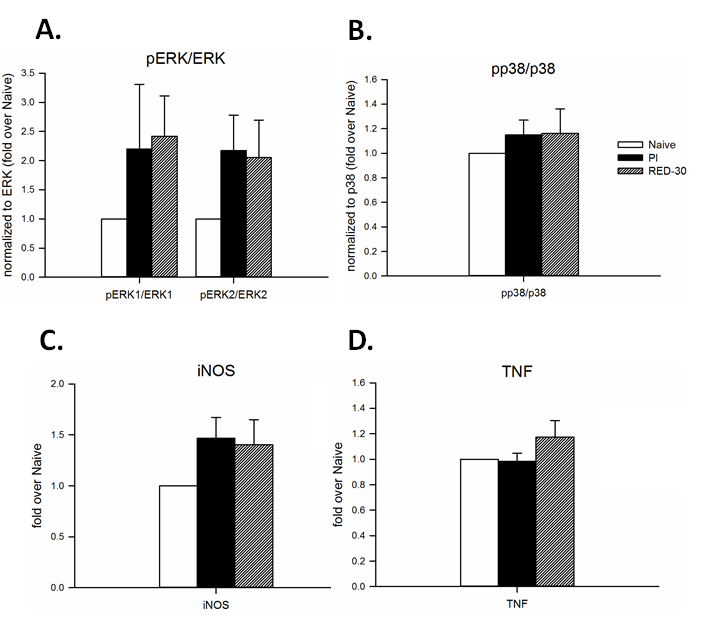

Supplement: Supplementary file 2 — High resolution image (TIFF 124 kb) [file 10103_2017_2367_MOESM1_ESM.tif]
